# Overview on postmenopausal osteoporosis and periodontitis: The therapeutic potential of phytoestrogens against alveolar bone loss

**DOI:** 10.3389/fphar.2023.1120457

**Published:** 2023-02-23

**Authors:** Putri Ayu Jayusman, Nurrul Shaqinah Nasruddin, Badiah Baharin, Nurul ‘Izzah Ibrahim, Haryati Ahmad Hairi, Ahmad Nazrun Shuid

**Affiliations:** ^1^ Department of Craniofacial Diagnostics and Biosciences, Faculty of Dentistry, Universiti Kebangsaan Malaysia, Kuala Lumpur, Malaysia; ^2^ Unit of Periodontology, Department of Restorative Dentistry, Faculty of Dentistry, Universiti Kebangsaan Malaysia, Kuala Lumpur, Malaysia; ^3^ Department of Pharmacology, Faculty of Medicine, Universiti Kebangsaan Malaysia Medical Centre, Kuala Lumpur, Malaysia; ^4^ Department of Biochemistry, Faculty of Medicine, Manipal University College, Melaka, Malaysia; ^5^ Department of Pharmacology, Faculty of Medicine, Universiti Teknologi MARA, Sungai Buloh Campus, Jalan Hospital, Sungai Buloh, Selangor, Malaysia

**Keywords:** periodontitis, postmenopausal osteoporosis, alveolar bone loss, phytoestrogens, estrogen deficiency

## Abstract

Osteoporosis and periodontitis are two major chronic diseases of postmenopausal women. The association between these two diseases are evident through systemic bone loss and alveolar bone loss. Both postmenopausal osteoporosis and periodontitis impose a considerable personal and socioeconomic burden. Biphosphonate and hormone replacement therapy are effective in preventing bone loss in postmenopausal osteoporosis and periodontitis, but they are coupled with severe adverse effects. Phytoestrogens are plant-based estrogen-like compounds, which have been used for the treatment of menopause-related symptoms. In the last decades, numerous preclinical and clinical studies have been carried out to evaluate the therapeutic effects of phytoestrogens including bone health. The aim of this article is to give an overview of the bidirectional interrelationship between postmenopausal osteoporosis and periodontitis, summarize the skeletal effects of phytoestrogens and report the most studied phytoestrogens with promising alveolar bone protective effect in postmenopausal osteoporosis model, with and without experimental periodontitis. To date, there are limited studies on the effects of phytoestrogens on alveolar bone in postmenopausal osteoporosis. Phytoestrogens may have exerted their bone protective effect by inhibiting bone resorption and enhancing bone formation. With the reported findings on the protective effects of phytoestrogens on bone, well-designed trials are needed to better investigate their therapeutic effects. The compilation of outcomes presented in this review may provide an overview of the recent research findings in this field and direct further *in vivo* and clinical studies in the future.

## 1 Introduction

• Osteoporosis is an age-related bone disease characterized by low bone mass and deterioration of bone tissue micro-architecture resulting in increased bone fragility and susceptibility to fracture (Sözen et al., 2017). It is an emerging geriatric condition in developing nations. It affects men and women of all races, but the prevalence of osteoporosis is higher among women compared to men (Clynes et al., 2020). The global increase in life expectancy, being 74 years for women has lead them to suffer from many debilitating diseases such as osteoporosis (Manyonda et al., 2020).
• The alveolar bone is the thick ridge of bone located on the jaw bones. It contains the tooth sockets which hold the teeth. Anatomically, these human bones are called the maxilla and mandible. Alveolar bone loss is one of the hallmarks of periodontitis. It causes weakening of the supporting structures of the teeth and predisposes to tooth mobility and loss. Postmenopausal osteoporosis is closely related to the development of periodontitis (Contaldo et al., 2020). Periodontitis, the sixth most prevalent disease worldwide, is multifactorial inflammatory disease mediated by host response and dysbiotic plaque biofilms, resulting in periodontal tissue destruction, alveolar bone loss and eventually tooth loss (Tonetti et al., 2017; Papapanou et al., 2018).
• Both osteoporosis and periodontitis are prevalent inflammation-associated bone disorders that have common features of bone resorption, being silent and asymptomatic (Mashalkar et al., 2018; Ayed et al., 2019). These diseases remain a major public health problem particularly in the aging population (Yu & Wang, 2022). It is projected that osteoporosis and periodontitis cases will increase as the population advances in age and is predicted to cause great health challenges (Wang and McClauley, 2016). As periodontitis leads to alveolar bone loss, tooth loss, edentulism and masticatory dysfunction, it could indirectly affect nutrition, impair quality of life and self-esteem of the affected individuals (Chang et al., 2020). Postmenopausal osteoporosis and periodontitis may impose huge socioeconomic impacts and healthcare costs (Brandão et al., 2012; Mohd-Dom et al., 2014; Mohd Dom et al., 2016). Both diseases share a number of risk factors such as age, smoking, alcohol consumption and diabetes, and common features of bone resorption that might require mutual concomitant management (Wang & McCauley, 2016).
• Anti-resorptive drugs such as biphosphonates are the commonly used pharmacological agents for the treatment of osteoporosis. Zoledronate is a long-acting bisphosphonate and most potent anti-resorptive drug that has been reported in the literature to have a positive effect on bone density in patients with osteoporosis. Apart from that, zoledronate could also improve periodontal disease and prevent tooth loss (Taguchi et al., 2019). Postmenopausal women on hormone replacement therapy (HRT) were found to have better natural teeth retention than those not receiving HRT (Han et al., 2016). However, the use of these treatment modalities is associated with unwanted side effects. Anti-resorptive agent biphosphonate may lead to renal toxicity, acute-phase reactions, gastro-intestinal toxicity, and osteonecrosis of the jaw (Ralston, 2015). The use of HRT is also associated with side effects and risks, including stroke, thromboembolism, vascular diseases and breast cancer. It was reported that to avoid the HRT adverse effects, women nowadays are shifting to herbal medicine, particularly for the prevention and treatment of menopause related symptoms (Gerbarg & Brown, 2016; Djapardy and Panay. 2022). The search of natural substances with promising results for the treatment of postmenopausal osteoporosis and periodontitis therefore is highly desirable.
• In this regard, phytomedicine or plant-based medicine with therapeutic and healing properties have gain scientific and clinical interest. Phytoestrogens are naturally occurring non-steroidal polyphenolic compounds that have structural and biological similarity to 17-β-estradiol, the main female sex hormone. Even though the affinity is lesser than that of endogenous estrogens, phytoestrogens can bind to estrogen receptors (ER) and exert anti-estrogenic or pro-estrogenic effects (Rowe and Barber, 2021). Most of phytoestrogens are also antioxidant and anti-inflammatory agents and these properties contribute to their distinguished therapeutic health effects Kładna et al., 2016. A growing body of evidence supported their therapeutic potential in preventing and treating several dysfunctions and diseases related to aging including menopausal symptoms and osteoporosis (Sirotkin & Harrath, 2014). In fact, phytoestrogens are used as a dietary supplement and as an alternative to HRT as they are believed to be safe and effective. Such properties turn these substances into promising targets for development as adjunctive preventive and therapeutic strategies for postmenopausal osteoporosis and periodontitis. In this review, we summarized the effects of the most studied phytoestrogens on bone health and screened phytoestrogens with the most promising alveolar bone protective effect in postmenopausal osteoporosis model with and without experimental periodontitis. This review may provide important insights for further *in vivo* and clinical studies of postmenopausal osteoporosis and periodontitis.

## 2 Association between postmenopausal osteoporosis and periodontitis

The association between postmenopausal osteoporosis and periodontitis has been reported extensively in epidemiologic and experimental studies (Luo et al., 2014; Juluri et al., 2015; Ayed et al., 2019). Though the mechanism of periodontitis in postmenopausal women has not been fully elucidated, an explicit understanding on the mechanistic link between the two diseases and their interplay is important for the prevention and management of these disorders, particularly in the elderly. The pathogenesis of postmenopausal osteoporosis involved the activation of systemic inflammation and dysregulation of immune response (Al-Daghri et al., 2017; Fang et al., 2022). Healthy bone continuously remodels through osteoblast-mediated bone formation and osteoclast-mediated bone resorption until the fourth to sixth decade of life when resorption exceeded the formation, causing a continuous loss of bone mass and a progressive decline in bone mineral density (BMD) (Kirk et al., 2020; Munoz et al., 2020). The cessation of ovarian function at menopause is one of the main causes of osteoporosis (Wu et al., 2021). Withdrawal of the protective effect of estrogen and immune cells alteration contribute to ongoing bone destruction in postmenopausal osteoporosis (Fischer and Haffner-Luntzer, 2022). The crosstalk between the immune system and the bone has been reported in the literature since the past decades (Takayanagi et al., 2000). Various immune cells interact with bone cells, the osteoblasts and osteoclasts, through cell-cell direct contact or *via* paracrine mechanisms (Fischer and Haffner-Luntzer, 2022). Immune cells including subtypes of T lymphocytes, B lymphocytes, macrophages, neutrophils and mast cells influence bone cells *via* factors including inflammatory cytokines, the interleukin (IL)-6 and tumor necrosis factor -α (TNF-α), osteoprotegerin/receptor activator of nuclear factor kappa-β ligand (OPG/RANKL) and other mediators, by increasing osteoblast apoptosis and stimulating osteoclastogenesis, thereby triggering bone loss during postmenopausal osteoporosis (Du et al., 2018; Zhang et al., 2020; Fischer and Haffner-Luntzer, 2022).

Bacterial dental plaque or biofilm is the primary etiological factor that dissociate periodontitis from osteoporosis. Though the pathogenesis and progression of periodontitis is primarily dependent on host interaction with the dysbiotic biofilm, the subsequent exacerbation of inflammatory response and its influence on bone homeostasis play crucial roles in both osteoporosis and periodontitis (Yu & Wang, 2022). Inflammatory response, the recruitment of polymorphonuclear neutrophils in particular, is the first line of defense against the invading periodontal pathogens in subgingival dental biofilm that is intended to eliminate the initial cause of tissue injury. As inflammation involve the activation of immune cells in the innate and adaptive immunity, the interplay between microbes and immune components initiate and propagate periodontal inflammation (Hajishengallis, 2014). The activation of lymphocyte and amplification of local inflammatory signaling cascade could stimulate RANKL signal, promoting osteoclastogenesis and inhibiting osteoblast lineage cells, thereby causing an uncoupling of bone remodeling process (Kawai et al., 2006; Pacios et al., 2015). These events are thought to be responsible for bone resorptive lesion and periodontal bone loss in periodontitis.

As described above, it is known that osteoporosis and periodontitis are closely related with inflammation and aging. Apart from inflammation, oxidative stress, is another major causative factor implicated in the pathogenesis and progression of these diseases. During aging, the accumulation of intracellular reactive oxygen species (ROS) and depletion of antioxidant enzymes lead to the elevation of oxidative stress in skeletal system (Manolagas, 2010). Excessive production of ROS, which is also responsible for elevation of immune cytokines could trigger the increase in osteoclastogenesis and osteoblast apoptosis as well as decrease in osteoblastogenesis. A population-based study suggested an interplay between oxidative stress and bone resorption that possibly underlies the development of postmenopausal osteoporosis (Cervellati et al., 2014). Menopause-related estrogen withdrawal increase the risk of postmenopausal osteoporosis basically by making the bone more vulnerable to oxidative injury. The theory that oxidative stress affects BMD was also supported by numerous clinical studies. Oxidant and antioxidant status imbalance in postmenopausal osteoporosis could affect the osteoclastic and osteoblastic activity. With regards to its involvement in periodontitis, oxidative stress has been linked to periodontal tissue destruction. ROS initially act as an antimicrobial defense system by killing the invaded pathogenic microorganism triggered by the infiltration of polymorphonuclear neutrophil (Wang, 2015). The generation of ROS is also considered as a “double-edge” sword as it helps to kill invading pathogen and it can be cytotoxic to host cells when the inflammatory response is exaggerated. Overproduction of ROS within the affected tissues leads to oxidant-antioxidant imbalance which then results in oxidative stress and pathological changes and consequently cause destruction of host tissues (Sczepanik et al., 2020). Hence, ROS is responsible for destruction of periodontal tissues and tooth loss in periodontitis. Due to these facts, periodontitis is also referred to as an inflammatory disease of oxidative stress.

The evolution of classification and diagnostic criteria for osteoporosis and periodontitis have been described and reported in the literature. Osteoporosis is determined by the measurement of BMD, expressed in terms of the number of standard deviations (SD) from the mean BMD of healthy individuals that matched to age and sex (Z-score), and the number of SD from the mean BMD of healthy young sex-matched individuals (T-score). According to WHO criteria, osteoporosis is present when BMD lies 2.5 SD or more below the BMD of young healthy women. Meanwhile, osteopenia or low bone mass is defined as BMD levels between one SD and 2.5 SD below the normal BMD (Lorentzon & Cummings, 2015). The most widely recognized tools used to measure BMD is dual energy x-ray absorptiometry (DXA). This technique is a standard non-invasive diagnosis approach that is reliably used worldwide to identify patients with low BMD due to its high precision and resolution but low radiation and cost. Additionally, quantitative ultrasound (QUS) methods have been used in the diagnosis and follow-up treatment in osteoporosis. Advances in computed tomography (CT) and magnetic resonance imaging (MRI) are promising in clinical and research settings in terms of capturing bone microarchitecture and also characterizing processes at the molecular level (Oei et al., 2016).

The diagnosis of periodontitis is primarily based on clinical evaluation whereby radiographs are used to confirm the clinical manifestation. The patient is considered to have periodontitis when there are at least four teeth with 4 mm probing depth in one or more sites, clinical attachment loss (CAL) up to 3 mm at the same site and presence of bleeding on probing (Ayed et al., 2019). The American Academy of Periodontology and the European Federation of Periodontology has modified the definition and classification framework for periodontitis based on staging and grading system (Tonetti et al., 2017). The staging refers to the severity and extent of periodontitis at present while grading refers to the rate of its progression. In clinical setting, the periodontal health can be evaluated by the measurement of CAL using probing pocket depths (PPD) and gingival recession but the reliability of this method is limited in terms of probing force, angulation, placement and tip diameter (Trombelli et al., 2018). Radiographic bone loss (RBL) should be used in cases if the CAL is unavailable (Tonetti et al., 2017). Loss of alveolar crestal height (ACH), oral hygiene simplified (OHI-S) and sites with bleeding on probing (BOP) percentage are among other important clinical parameters in the evaluation of periodontal status (Qi et al., 2021). Apart from that, identification of cavities and periodontal lesions, maxillary sinusitis and other lesions in the oral and maxillofacial field as well as osteoporosis can be done with the use of computer-aided diagnosis (CAD) (Wang and McCauley. 2016).

Tooth loss, periodontal disease, ill-fitting or loose dentures and severe bone loss around the teeth, are among the early indicators of osteoporosis detected in the oral cavity (Ayed et al., 2019). In the clinical setting, patients at risk can be identified by a dentist from medical history, clinical examination and radiographic findings while patient’s osteoporosis status can be obtained by further determination of BMD together with the evaluation of dental radiography. Knowledge on the relationship between postmenopausal osteoporosis and periodontitis, as well as sufficient clinical and radiographic information would enable dentists to play their role in early diagnosis and screening of osteoporosis in postmenopausal women. Regardless of gender, patients with osteoporosis may have two-fold increase in the risk of periodontitis. Though osteoporosis is not an etiologic factor in periodontal disease, osteoporotic women presented a higher risk and greater severity of periodontitis than men (Pepelassi et al., 2012; Manjunath et al., 2019). This association suggested that patients with osteoporosis should be evaluated for periodontal health and *vice versa*. Efforts towards the prevention of periodontal disease in patients at risk of osteoporosis particularly in postmenopausal women would be enviable. Although the mechanisms underlying this association has not been not fully elucidated, information collected from the literature strongly explained the association.

## 3 Estrogen deficiency and inflammatory response in alveolar bone loss

Estrogen is a steroid hormone that is not only responsible for female sexual characteristics development but also has other non-reproductive physiological roles. It is one of the key regulators of bone metabolism with significant influence on skeletal growth and homeostasis in both women and men. Estrogens act directly on osteoblasts, osteocytes, immune cells and other cells *via* the ER found on these bone cells (Bord et al., 2001; Braidman et al., 2001; Crusodé-Souza et al., 2009). In general, ER signaling pathway activation stimulated differentiation of osteoblast and suppressed osteoclastic activity (Manolagas, 2000). Estrogen also prevented apoptosis of osteocytes and its anti-apoptotic effect is related to their autophagy regulation in osteocytes (Florencio-Silva et al., 2018). Estrogen deficiency increased osteoclastogenesis, prolonged osteoclast lifespan and increased the rate of bone turnover, causing accelerated resorption than formation (Manolagas, 2000; Manolagas et al., 2013). A recent study showed that estrogen deficiency decreased autophagy and increased apoptosis in alveolar process osteocytes, whereby estrogen replacement enhanced osteocyte viability simply by inhibiting apoptosis and maintaining autophagy in these cells (Florencio-Silva et al., 2018).

Apart from its direct effects on bone cells, research has revealed estrogen regulated bone hemostasis through its influence on the immune system and on oxidative stress. In essence, the proinflammatory cytokines, IL-1, IL-6, TNF-α, granulocyte macrophage colony-stimulating factor, macrophage colony-stimulating factor (M-CSF), and prostaglandin-E_2_ (PGE_2_) played a significant role in bone metabolism by increasing bone resorption (Riggs, 2000). These proinflammatory cytokines particularly IL-1, IL-6 and TNF-α were considered as osteoclastogenic bone resorption-inducing cytokines (Scheidt-Nave et al., 2001). Activation of inflammatory cascades due to estrogen deficiency led to increased production of M-CSF and RANKL. The binding of M-CSF to its receptor stimulated the proliferation of osteoclast and the survival of its precursors as well as mature osteoclasts. The binding of RANKL to RANK receptors stimulated differentiation and activity of osteoclast and prevented their apoptosis (Feng et al., 2019). In osteoporotic patients, the increased in systemic levels of IL-6 could predict BMD change and fracture rate (Barbour et al., 2014). A significant elevation in IL-6 as well as TNF-α were also observed in ovariectomized (OVX) animals compared to the SHAM group (Eminov et al., 2018; Delgobo et al., 2019). Apart from the increase in IL-6 expression, OVX rats also showed increased RANKL and to a lesser extent, OPG expression. OVX rat is a suitable model for studying postmenopausal bone loss as it mimics the decline in endogenous estrogen production by the ovaries during menopause (Johnston & Ward, 2015). This model resembles deterioration of bone tissue in the hip and spine in postmenopausal osteoporosis and hence it can also be used to study mineral and structural changes in alveolar bone.

Alveolar bone loss due to periodontitis is a frequent complication in postmenopausal women suffering from osteoporosis due to estrogen deficiency (Tanaka et al., 2020). As described earlier, changes in hormone level particularly estrogen have impact on systemic bone homeostasis and inflammatory response. Following menopause, estrogen levels in the circulation fall drastically as the production by the ovaries cease. Estrogen reduced osteoclast activity and prevented apoptosis of osteocytes, and for that reason rapid decline in estrogen may lead to systemic bone loss due to disruption of bone homeostasis (Grover et al., 2014). Other than bones, estrogen receptors can also be found in the oral mucosa, gingiva and salivary glands. ER-β is the predominant ER present in the gingival epithelium (Valimaa et al., 2004). Therefore, hormone fluctuations may also contribute to changes in the oral cavity and acceleration of inflammatory response (Jafri et al., 2015). Upregulation of immune cells and osteoclasts due to estrogen deficiency eventually result in a greater production of bone-resorbing cytokines. The increase in inflammatory cytokines and other factors in the circulation may not only have impact on systemic bone remodeling but also may locally compromise the tissue response to periodontal disease (Golub et al., 2006).

Estrogen deficiency in postmenopausal women or in experimental OVX rodents has been markedly linked to alterations in trabecular and cortical bone including the alveolar bone of mandible (Johnston & Ward, 2015; Jonasson et al., 2018). Studies also showed that estrogen deficiency aggravates the severity of experimental periodontitis (Anbinder et al., 2016). Clinically, postmenopausal women with osteoporosis and concurrent periodontitis have been found to exhibit an exaggerated response to dental plaque, higher periodontal attachment loss and a significant reduction of alveolar bone compared to healthy women (Singh et al., 2014). These changes are evident through the assessment of periodontal health such as increased gingival bleeding and periodontal pocket depth, decreased BMD of the alveolar crestal and subcrestal bone, loss of dentoalveolar bone height, or loss of clincial attachment and tooth (Marjanovic et al., 2013; Jang et al., 2015; Juluri et al., 2015; Savić Pavičin et al., 2017).

**FIGURE 1:**
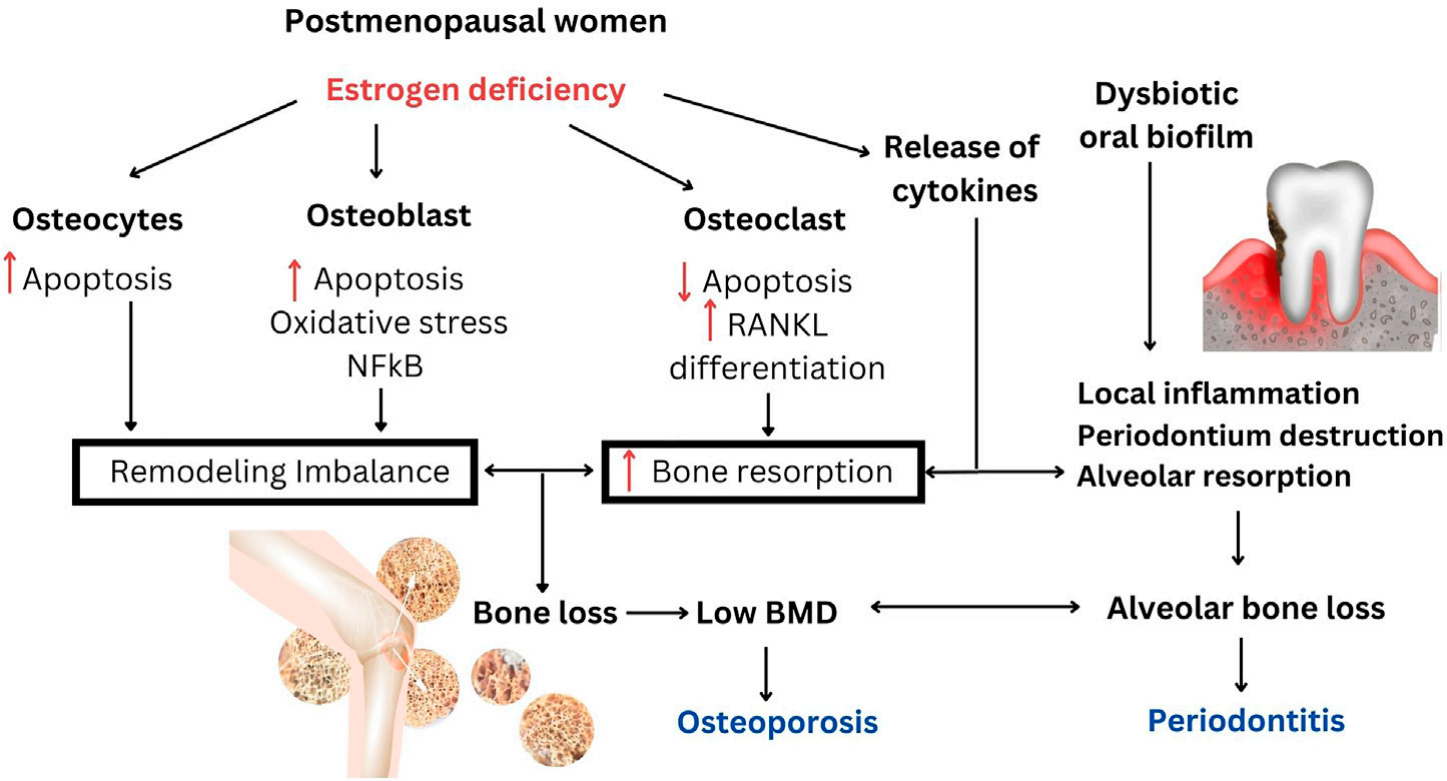
The impact of estrogen deficiency and the association between postmenopausal osteoporosis and periodontitis. Estrogen deficiency is responsible for the increase in systemic bone resorption and remodeling imbalance that would also accelerate the increase in alveolar bone resorption. Loss of BMD in postmenopausal osteoporosis may be responsible for increasing alveolar bone loss in periodontitis. Local inflammation in periodontitis may activate systemic osteoclastogenic cytokines and further aggravates bone loss.

It was hypothesized that osteoporosis could accelerate alveolar bone resorption and bone loss in periodontitis because the loss of alveolar BMD allowed deeper bacterial penetration into the enlarged periodontal space. In response to local inflammation, alveolar resorption could be further amplified and accelerated as focal infection of the periodontium may also release inflammatory cytokines into the system (Barbato et al., 2015). Additionally, overexpression of proinflammatory cytokines with osteoclastic activity occured in both osteoporosis and periodontitis (Barbato et al., 2015; Inchingolo et al., 2020). The impact of estrogen deficiency and the association between postmenopausal osteoporosis and periodontitis are shown in the schematic diagram (Figure 1).

## 4 Therapeutic potential of phytoestrogens

Phytoestrogens are generally divided into four major groups: isoflavones, stilbenes, coumestans and lignans. Isoflavones are the most widely used and studied phytoestrogens. They are found primarily in soybeans and other legumes, which constitute the major dietary source of phytoestrogens in Asian communities (Rowe & Baber, 2021). Genistein and daidzein are the two well-characterized isoflavones that have also been shown to have estrogenic potential. Resveratrol is the most common and the main dietary source of phytoestrogenic stilbenes. Its estrogenic activity is dependent on the two isomers, cis and trans. Trans has been reported to have higher estrogenic activity (Desmawati & Sulastri, 2019). Coumestans are biosynthetically related to isoflavones but only a small number of coumestans have shown estrogenic activity (Poluzzi et al., 2014). Lignans, the major dietary source in Western diets, are mostly derived from fruit, vegetables, legumes and whole grains. Lignan dimers that are not estrogenic themselves can be converted by gut microflora to mammalian lignans, the enterodiol and enterolactone which are estrogenic (Cornwell et al., 2004).

Phytoestrogens have been extensively studied for their potential role to prevent and treat diseases related to aging such as menopausal symptoms and skin aging, cardiovascular, neurodegenerative, immune and metabolic diseases and cancer. Recent systematic review and meta-analysis reported that consumption of low doses of phytoestrogen (25 mg/d ≤ dose ≤100 mg/d) for a long-term duration were effective in relieving depression symptoms in postmenopausal women (Li et al., 2021). Some reported adverse effect associated to phytoestrogens in menopausal women however are not yet clear and required more supportive evidence from high-quality randomized control trial (RCT) studies. Animal studies showed that high-dose administration of phytoestrogens (equol and *puerarian mirica*) modulated female reproductive system by enhancing the levels of serum luteinizing hormone and reducing urinary follicular stimulating hormone in ovariectomized rats and cynomolgus monkeys respectively (Rachoń et al., 2007; Trisomboon et al., 2007). Exogenous estrogen-like molecules could promote reproductive function and it also could possibly destroy reproductive processes (Sirotkin & Harrath, 2014). Nevertheless, no adverse effects of phytoestrogens on human reproduction has been reported yet. In a RCT, supplemention of 100 mg isoflavones-rich, concentrated soy extract daily to postmenopausal women for 6 months has improved their skin health by increasing epithelial thickness, number of elastic and collagen fibres (Accorsi-Neto et al., 2009).

Soy genistein has been proposed to have a promising therapeutic effect for metabolism improvement and treatment of metabolic disorder (Behloul & Wu, 2013). In an RCT, 250 mg per day of genistein administered to non-alcoholic fatty liver patients for 2 months demonstrated reduction in insulin levels, indicating the ability of phytoestrogen to modulate human endocrine system (Amanat et al., 2018). Supplementation of soy protein with 66 mg isoflavones for 6 months has been found to significantly improve cardiovascular disease risk markers in women during the early menopause (Sathyapalan et al., 2018). A meta-analysis of epidemiological studies has found that the intake of soy isoflavones by pre- and post-menopausal women in Asian countries could lower the risk of breast cancer (Chen et al., 2014). Several other studies have shown the potential effect of phytoestrogen consumption in reducing the risks of lung cancer (Shimazu et al., 2011), stomach cancer (Ko et al., 2013), prostate cancer (He et al. 2015; Hwang et al., 2009), endometrial and ovarian cancer (Bandera et al., 2009; Qu et al., 2014).

The therapeutic potential described above indicated that phytoestrogens possess beneficial effect on the health of various organs and systems at different doses. Though the main mechanism of action of phytoestrogens is due to ER binding, their antioxidant, anti-inflammatory and other properties could also contribute to their pro-health effects. For example, the interaction of phytoestrogens with ER as well as their antioxidant properties might contribute to its neuroprotective effects (Gorzkiewicz et al., 2021). Several studies showed that different forms of soybean including soy isoflavones have antibacterial activity against oral microbes (Lee & Kim, 2006; Wang et al., 2010; Laodheerasiri and Horana Pathirage, 2017; Choo et al., 2020; How et al., 2020). Antimicrobial activity of phytoestrogens may be useful for the treatment and prevention of periodontal disease as periodontitis is known to have bacterial cause. Dental plaque and polymicrobial infections play a pivotal role in the initiation of periodontitis. For this reason, elimination or controlling the bacteria could be a beneficial approach in managing periodontal diseases.

## 5 Phytoestrogens and bone

In view of its role as the key regulator of bone metabolism, it has been hypothesized that phytoestrogens exerted bone health effects through their estrogenic potential, usually by binding to estrogen receptors (Chiang and Pan. 2013). As described earlier, soy isoflavones is one of the most widely studied phytoestrogens and they have received considerable attention in the management of postmenopausal bone loss. In the last decades, numerous clinical studies have been conducted in a wide range of populations including Western and Asian counterparts using different types of isoflavones preparations. Observational studies showed that women who consume higher amounts of soy foods have lower risk of postmenopausal fracture (Setchell & Lydeking-Olsen, 2003; Messina et al., 2004; McCarty, 2006) and as a consequent many clinical trials have attempted to evaluate the protective role of soy isoflavones on bone health (Lagari & Levis, 2013). Reported findings on the skeletal effects of isoflavones from clinical trials however are inconsistent due to the variation of study design and length of intervention, study population, dose and types of isoflavones preparation. The inconclusive results from human clinical trials are generally contributed by their heterogeneity and poor quality (Rowe & Baber, 2021).

A systematic review of RCT in 2016 compiled 23 eligible studies that ranged from 7 weeks to 3 years of interventions, which mainly assessed the effectiveness of phytoestrogens intervention through the measurement of whole body or regional BMD or bone mineral content, T-scores and bone metabolism biomarkers during the menopause transition (Abdi et al., 2016). Though there were controversial reports about changes in BMD, different types of soy isoflavones extracts (including genistein and daidzein), dietary products containing different amounts of phytoestrogens and red clover extract may have beneficial effects on bone health in postmenopausal women. A recent meta-analysis and systemic review of RCT concluded that isoflavones could be beneficial in preserving BMD and reducing bone resorption in premenopausal and postmenopausal women (Lambert et al., 2017). This could be linked to the effect of phytoestrogens principally as antiresorptive agents rather than their potential in bone formation. The use of isoflavones aglycones with well-controlled, standardized and defined isoflavones interventions revealed greater efficacy in treating BMD loss in estrogen-deficient women compared to glycosides and less well-defined isoflavones formulation. One year intake of novel red clover extract rich in isoflavones aglycones and probiotics has been found to potently attenuated BMD loss and improved bone turnover in postmenopausal osteopenic women (Lambert et al., 2017).

Although human clinical trials are inconsistent and some reported negative findings, numerous *in vitro* and animal studies on phytoestrogens revealed encouraging bone sparing effects. Phytoestrogen could promote osteogenesis by specifically targeting osteoblast and osteoclast. The administration of phytoestrogens such as isoflavones and flavonoids promoted bone formation, which stimulate the expression of osteogenic markers such as Runx2, ALP, osteocalcin, type 1 collagen (COL-1), osteopontin, and morphogenetic protein-2 (BMP-2) for osteoblast differentiation and bone matrix mineralization (Ramesh et al., 2021; Ortiz et al., 2022; Sekaran et al., 2022). Additionally, by virtue of its similar structure to 17β-estradiol in conformational binding to estrogen receptors, phytoestrogen has capability to reciprocally affect osteogenic *versus* adipogenic differentiation of mesenchymal stem cells (MSCs) in a dose dependent manner. For instance, phytoestrogen stimulated osteoblast differentiation from MSCs *via* the activation of important signalling pathways such as Smad, Wnt/β-catenin and Sirt1 pathways (Schilling et al., 2014; Gorabi et al., 2019; Khezri et al., 2021; Wang et al., 2021). Concomitantly, there is evidence that phytoestrogen suppressed the adipogenic differentiation signalling pathway including PPAR and C/EBP pathways in a dose dependent manner (Schilling et al., 2014; Ahmed 2014).

In addition, the process of bone resorption and formation are closely coupled through the RANKL/OPG system. The integrity of the skeleton is maintained through bone formation followed by a balanced cycle of bone resorption (Narducci et al., 2011; Sharma et al., 2022). Intriguingly, phytoestrogen was able to regulate the RANKL/OPG system, thereby playing an important role in the pathogenesis of osteoporosis (Ni et al., 2023; Hooshiar et al., 2022; Zakłos-Szyda et al., 2020). Furthermore, phytoestrogen exerted antiresorptive activities by suppressing the expression of osteoclast differentiation markers such as matrix metalloproteinase 9 (MMP9), cathepsin-K and tartrate-resistant acid phosphatase (TRAP) *via* downregulating the activation of NF-κB and MAPK signaling pathways (Xu et al., 2022; Tanaka et al., 2020). These findings demonstrated that phytoestrogen treatments could inhibit osteoclast activation and therefore have therapeutic potential for osteoporosis.

The primary endpoints that generally measured following hormone ablation in *in vivo* studies include, trabecular bone and/or cortical bone mass, BMD and mechanical strength. Changes in bone turnover markers and uterine weight were also measured. Phytoestrogens significantly increased both trabecular and/or cortical bone volume in OVX-induced bone loss with no uterotrophic effect in animal models (Inchingolo et al., 2020; Long et al., 2022). Phytoestrogen was also shown to significantly decreased urinary excretion of deoxypyridinoline (DPD), which is one of the bone resorption markers (Li et al., 2021; Song et al., 2016). Furthermore, phytoestrogen exerted antioxidant effects as shown by the increased expression of antioxidant enzymes such as superoxide dismutase (SOD) and gluthathione (GSH), that serve to scavenge the excess free radicals. This antioxidative effect of phytoestrogens also inhibit osteoclast differentiation (Oršolić et al., 2022). Overall, findings from *in vivo* studies are in line with *in vitro* studies, which demonstrated the bone-conserving effects of phytoestrogens in reducing bone loss due to estrogen deficiency.

### 5.1 Effects of phytoestrogens on alveolar bone loss

Being a systemic disease, the manifestation of bone loss in osteoporosis is not only evident in vertebrae and appendicular skeleton but also in alveolar bone (Muslita et al., 2012). For this reason, osteoporosis is expected to accelerate alveolar bone resorption caused by periodontitis (Guiglia et al., 2013). Additionally, osteoporosis results in an increase in certain inflammatory cytokines which are also affected in the progression of periodontitis. Oxidative stress is also an indicator of periodontitis development, which is evident by the accumulation of ROS (Kanzaki et al., 2017). Studies on the potential skeletal effects of phytoestrogens in postmenopausal osteoporosis at the preclinical (*in vitro* and in animal models) and clinical level have been substantially reported in the literature. To the best of our knowledge, studies on the effect of phytoestrogens on postmenopausal osteoporosis with periodontal disease in human are still lacking. Nonetheless, several animal studies have been carried out recently to evaluate the protective effects of phytoestrogens against alveolar bone loss in postmenopausal osteoporosis with or without experimental periodontitis.

Table 1 summarizes the effect of phytoestrogens against alveolar bone loss. In a recently published study, oral administration of soy isoflavones was found to alleviate experimental periodontitis in estrogen-deficient rats as revealed by the increased expression of tight junction proteins in the gingiva, reduced proinflammatory cytokines, IL-17 and ROS levels (Liu et al., 2022). Attenuation of alveolar bone loss was observed through micro-CT and histologic observation. Experimental periodontitis in OVX rats was established by silk ligature and inoculation with *Porphyromonas gingivalis,* a Gram-negative anaerobe, which is one of the well-characterized periodontal pathogens involved in periodontitis. *P. gingivalis* possibly modulate the immune response through the inactivation of certain cytokines (Baek et al., 2015). An *in vitro P. gingivalis* infection model was also used to determine whether grainyhead-like 2 (GRHL2), the epithelial transcription factor and ER-binding partner (He et al., 2020) is required by soy isoflavones to enhance the oral epithelial barrier. It was found that the enhancement of oral gingival epithelial barrier function by soy isoflavones treatment was partially dependent on GRHL2 (Liu et al., 2022).

**TABLE 1:** Summary findings on the therapeutic potential of phytoestrogens against alveolar bone loss.

Phytoestrogen	Summary findings	Author
Soy Isoflavones	Soy isoflavones increased the expression of tight junction proteins, and reduced IL-17 level and alveolar bone loss, alleviating periodontitis in ovariectomised rats	Liu et al. (2022)
Diosgenin	Diosgenin significantly reduced TNF-α and osteocalcin expression. Results from circRNA profile and the circRNA-miRNA-mRNA network demonstrated that the potential mechanism of diosgenin to inhibit osteoclastogenesis by regulating the expression of Wnt, PI3K, RANK/RANKL or osteoclastogenic cytokine pathways	Zhang et al. (2020)
Diosgenin	Diosgenin significantly reduced the level of TRAP and increased the level of ALP. Diosgenin promoted the bone formation process by increasing Smad4, Smad8, and beta-catenin/Tcf, osterix, ALP and OPN and inhibited two potent stimulators of osteoclastogenesis TNF-α and IL-1 β and their receptors, IL-1R and TNF-R1. mRNA expression of TRAP in alveolar bone was shown to be downregulated after a 12-week diosgenin treatment	Zhang et al. (2018)
Genistein	Histological and µCT analyses demonstrated that genistein administration decreased distance between the CEJ and the apex of the alveolar bones. Genistein significantly reduced the level of TRAP, COX-2 and ICAM expression in the inflamed region of mice with periodontitis	Bhattarai et al. (2017)
Genistein	Genistein administration prevented alveolar bone loss significantly induced by ligature placement (about 74%). Genistein administration also increased microstructural parameters of trabecular bone, including Tb.Th, Tb.Sp, bone BMD and structure model index	Choi et al., 2016

IL, interleukin; TNF-α, tumor necrosis factor; RANKL, receptor activator of nuclear factor kappa-ligand; OPN, osteopontin; ALP, alanine phosphatase; TRAP, tartrate-resistant acid phosphatase; µCT, micro-computed tomography; COX-2, clyclooxygenase; ICAM, intercellular adhesion molecule; Tb.Th, trabecular thickness; Tb.Sp, trabecular separation; BMD, bone mineral density; SMI, structure model index; BMD, bone mineral density.

Earlier studies showed that genistein, a major subclass of isoflavones found in soybean was protective against periodontitis-induced alveolar bone loss (Choi et al., 2016; Bhattarai et al., 2017). Genistein was found to significantly attenuated *Prevotella intermedia* LPS-induced production of inducible nitric oxide synthase (iNOS) and IL-6 coupled with the decreased in their mRNA expression in RAW264.7 cells. In experimental animal, alveolar bone height and bone volume fraction were decreased and microstructural parameters of trabecular bone were improved with administration of genistein (Choi et al., 2016). These findings were in line with a study by Bhattarai et al. (2017) that also showed a reduction in LPS-induced alveolar bone loss with genistein administration. Additionally, genistein significantly prevented osteoclast differentiation by suppressing the expression of osteoclast-specific molecules in NFκB ligand- or LPS-stimulated macrophages. Apart from its inhibitory effect on osteoclast activation, the protection against LPS-mediated stresses by genistein was indicated by the reduction of mitochondrial impairment and ROS accumulation, which lead to the reduction of periodontal damage. The reported antioxidant and anti-osteoclastic potential of phytoestrogen genistein might be protective against alveolar bone loss in postmenopausal osteoporosis condition.

**FIGURE 2:**
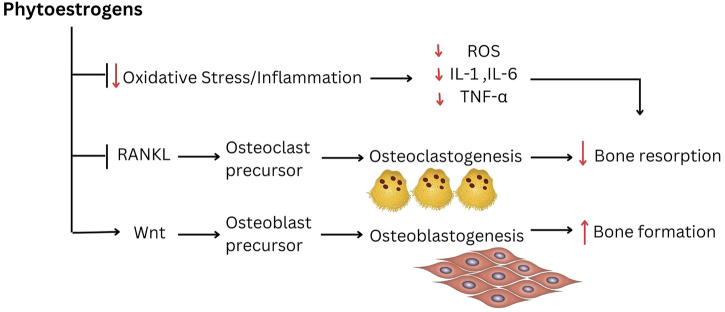
The possible molecular mechanism underlying the protection against alveolar bone loss by phytoestrogens.

A study done by Zhang et al. (2018) has found that 12-week oral treatment with diosgenin, a natural steroidal saponin and a phytoestrogen, suppressed alveolar bone loss in OVX rats by promoting bone formation. Though the effects of estradiol valerate on alveolar bone volume was greater than diosgenin, both treatments showed reduced alveolar bone loss compared to OVX rats as indicated by 3-D bone microstructure analysis and histological observation. The protective role of diosgenin on bone loss was also described earlier in the peripheral skeletal of OVX rats (Zhang et al., 2014; Folwarczna et al., 2016). The bone protective effect of diosgenin might be associated with the modulation of RANKL/OPG ratio (Zhang et al., 2014). As with other phytoestrogens, diosgenin may be one of the sparse compounds that have the potential to increase bone formation and inhibit bone resorption. In this study, lncRNA and mRNA profiles were evaluated using a microarray to confirm the anti-osteoporotic effects of diosgenin on alveolar bone. Diosgenin may have exerted this effect by increasing the Wnt and BMPs pathways, the two recognized signaling pathways that regulate the osteogenic differentiation of mesenchymal stem cells or preosteoblasts (Lin and Hankenson, 2011; Marcellini et al., 2012). This finding was further supported by another study by Zhang et al. (2020). The study revealed that anti-bone loss action of diosgenin on alveolar bone was attributed by the regulation of important molecules expression in the Wnt, P13K, RANK/RANKL or osteoclastogenic cytokine pathways. The possible molecular mechanisms underlying the protection against alveolar bone loss by phytoestrogens are summarized in Figure 2.

## 6 Conclusion

Association between postmenopausal osteoporosis and periodontitis has long been postulated though its causal relationship is yet to be determined. *In vitro* and animal studies have demonstrated phytoestrogens favorable effects on skeletal health. Phytoestrogens may exert their bone protective effect by inhibiting bone resorption and promoting bone formation. Phytoestrogens mainly isoflavones, may offer protection against alveolar bone loss in postmenopausal osteoporosis condition. Well-designed clinical trials are needed to determine the therapeutic potential of phytoestrogen on skeletal health particularly in postmenopausal women. Phytoestrogens can be potentially developed as adjunctive preventive and therapeutic cost-effective strategies in the treatment and prevention of bone loss in postmenopausal osteoporosis with periodontitis.
